# Psychological distress after esophageal cancer surgery and the predictive effect of dispositional optimism: a nationwide population-based longitudinal study

**DOI:** 10.1007/s00520-021-06517-x

**Published:** 2021-09-04

**Authors:** Yangjun Liu, Erik Pettersson, Anna Schandl, Sheraz Markar, Asif Johar, Pernilla Lagergren

**Affiliations:** 1 Department of Molecular Medicine and Surgery, Karolinska Institutet, Karolinska University Hospital, Stockholm, Sweden; 2 Department of Medical Epidemiology and Biostatistics, Karolinska Institutet, Stockholm, Sweden; 3 Department of Anaesthesiology and Intensive Care, Södersjukhuset, Stockholm, Sweden; 4 Department of Surgery and Cancer, Imperial College London, London, UK; 5 Nuffield Department of Surgery, University of Oxford, Oxford, UK

**Keywords:** Anxiety, Depression, Esophageal cancer, Optimism, Psychological distress, Survivorship

## Abstract

**Purpose:**

To examine the trajectory of psychological distress from 1 to 2 years after esophageal cancer surgery, and whether dispositional optimism could predict the risk of postoperative psychological distress.

**Methods:**

This Swedish nationwide longitudinal study included 192 patients who had survived for 1 year after esophageal cancer surgery. We measured dispositional optimism with the Life Orientation Test-Revised (LOT-R) 1 year post-surgery and psychological distress with the Hospital Anxiety and Depression Scale 1, 1.5, and 2 years post-surgery. Latent growth curve models were used to assess the trajectory of postoperative psychological distress and to examine the predictive validity of dispositional optimism.

**Results:**

One year after surgery, 11.5% (22 of 192) patients reported clinically significant psychological distress, and the proportion increased to 18.8% at 1.5 years and to 25.0% at 2 years post-surgery. Higher dispositional optimism predicted a lower probability of self-reported psychological distress at 1, 1.5, and 2 years after esophageal cancer surgery. For each point increase in the LOT-R sum score, the odds of psychological distress decreased by 44% (OR, 0.56; 95% CI, 0.40 to 0.79).

**Conclusion:**

The high prevalence and longitudinal increase of self-reported psychological distress after esophageal cancer surgery indicate the unmet demands for timely psychological screening and interventions. Measuring dispositional optimism may help identify patients at higher risk of developing psychological distress, thereby contributing to the prevention of postoperative psychological distress.

**Supplementary Information:**

The online version contains supplementary material available at 10.1007/s00520-021-06517-x.

## Introduction

Esophageal cancer is the seventh most common cancer globally [1]. It carries poor overall 5-year survival rate (< 20%) [2] and ranks the sixth leading cause of cancer-related death worldwide [1]. The mainstay of curative treatment, esophagectomy, is a highly invasive operation, which improves survival but entails high risk of postoperative complications and substantially impaired health related quality of life (HRQL) [3]. The life-threatening cancer diagnosis and major surgery are traumatic stressors for patients, leading to increased symptoms of psychological distress before and after esophageal cancer surgery [4–6]. Based on a prospective hospital-based cohort study carried out in London, 33%, 28%, and 37% of patients had anxiety prior to surgery, at 6 and 12 months after surgery, respectively [5]. Contrary to the stable trajectory of anxiety symptoms, depression symptoms first increase and then level off [5]. Prior to surgery, 20% of patients reported depression, and the proportion increased to 27% and 32% at 6 and 12 months after surgery, respectively [5]. However, the trajectory of psychological distress after 1 year post-surgery remains unclear. Revealing this trajectory may help healthcare providers and patients build proper expectations about postoperative recovery, thus taking timely measures to improve psychological adjustment.

Previous studies have identified some risk factors for psychological distress including younger age, female sex, cohabitating status, low education level, and tumor histology [7, 8]. However, patients with similar characteristics in these aspects still reported varied psychological status, which suggests that other factors, such as personality traits, may also play an important role in the psychological adjustment after esophageal cancer surgery.

Dispositional optimism is a personality trait referring to the expectation that positive rather than negative outcomes will happen in the future [9]. It has been shown that higher dispositional optimism is associated with a lower risk of developing psychological distress in patients with heart disease or other subtypes of cancer such as breast cancer, urogenital cancer, head and neck cancer, and oral cavity cancer [10–14]. However, whether this association exists in patients with esophageal cancer remains unknown. Clarifying the predictive effect of dispositional optimism on psychological distress may help identify high-risk patients and contribute to the implementation of timely and personalized interventions. In addition, given that dispositional optimism could be increased through psychological interventions such as Best Possible Self exercise and Cognitive Behaviour Therapy [15], it may be a potential intervention target to partly prevent psychological distress after esophageal cancer surgery.

In this study, we aim to explore the trajectory of psychological distress from 1 to 2 years after esophageal cancer surgery, and also to examine whether dispositional optimism predicted this trajectory.

## Methods

### Study design and data collection

Data for this longitudinal study is from a prospective, ongoing Swedish-nationwide cohort study entitled Oesophageal Surgery on Cancer patients—Adaptation and Recovery (OSCAR). Detailed description of the OSCAR study can be found elsewhere [16, 17]. In brief, it includes patients undergoing esophagectomy for cancer in Sweden from January 1, 2013 and onwards. Potential patients are identified by contacting pathology departments at eight hospitals performing esophagectomy in Sweden. At 1 year after esophageal cancer surgery, all survivors are invited to participate in the study. Patient-reported outcomes are collected from 1 year until 5 years after surgery through personal interviews and mailing paper questionnaires. Patients’ demographics are retrieved from the national health data registries and Swedish Longitudinal Integration Database for Health Insurance and Labor Market Studies. Clinical data are collected from medical charts, the Swedish Patient Registry and the Swedish Cancer Registry. Mortality information is obtained through linkage to the Swedish Register of the Total Population and the Swedish cause of death register. The study was approved by the Regional Ethical Review Board in Stockholm, Sweden (diary number 2013/844–31/1, 2015/2142–32, 2016/1696–32/1, 2017/1301–32, 2018/1447–32, 2019–04,289 and 2020–01,304) and written consents were obtained from all participants before inclusion.

### Study participants

Between January 1, 2013 and February 28, 2018, 647 patients underwent esophagectomy for cancer in Sweden. Among them, 154 patients died within 1 year post-surgery and 86 patients lacked valid contact information. Thus, 407 patients were invited to participate in the OSCAR study; 265 (65%) patients consented and attended the first (1-year) interview. Nonparticipation was mainly related to unwillingness, severe illness, and cancer recurrence [16]. We further excluded patients who died within 2 years after surgery (n = 49), with histories of psychological disorders (n = 5), with dysphasia confirmed by pathological histology (n = 3), and with missing data in clinical information, patient-reported outcomes and/or sociodemographic information (n = 16). Thus, the final analysis included 192 patients; of these, 170 and 156 patients answered the 1.5- and 2-year follow-up questionnaires, respectively.

### Psychological distress

Psychological distress was measured repeatedly at 1, 1.5, and 2 years after surgery using the Hospital Anxiety and Depression Scale (HADS) [18, 19], which is a well-validated and widely used questionnaire consisting of anxiety and depression subscales [18, 19]. Both subscales contain seven questions and each question is scored on a four-point Likert scale ranging from 0 to 3, with a higher score representing more severe symptoms. The Cronbach’s alpha for the anxiety and depression subscales were 0.83 and 0.74, respectively. The correlation between the two subscales was 0.57. A score ≥ 8 on each subscale is indicative of a “possible-probable” case of anxiety or depression, respectively [18]. Because anxiety and depression usually coexist [20], we treated psychological distress as a binary outcome and classified patients scoring ≥ 8 on either subscale as having clinically significant psychological distress.

### Dispositional optimism

Dispositional optimism was assessed at 1 year after surgery with the validated Swedish version of Life Orientation Test-Revised (LOT-R) [21, 22]. It consists of three positively worded items and three negatively worded items [21, 22]. Patients were asked to report their agreement with each five-point Likert item, ranging from 0 (“strongly disagree”) to 4 (“strongly agree”) [22].

The dimensionality of LOT-R remains controversial and no study has assessed its factor structure among patients with esophageal cancer. Therefore, we conducted a series of confirmatory factor analyses (see Supplementary Content 1). In the best fitting model, the loading of the first negatively worded item was negative, even though its score had been reversed to account for the negative wording. Moreover, this item had bimodal response distribution and equivocal correlations with both positively worded items and negatively worded items, indicating that a substantial proportion of the participants most likely misread it. Thus, we removed this item and reassessed the factor structure based on the remaining five items. The model assuming one factor (dispositional optimism) with correlated errors between the two reversed negatively worded items was adopted because it had the best model fit and strong theoretical base. The internal reliability estimated by McDonald’s omega for this model was 0.49 [95% bootstrapped confidence interval (CI), 0.31 to 0.62]. We summed up the five items, of which the two negatively worded items were reversed. A higher LOT-R sum score represents higher dispositional optimism.

### Statistical analysis

We compared the LOT-R sum score between patients with different sociodemographic and clinical characteristics using Student’s t-test and ANOVA. Latent growth curve model with maximum likelihood estimator and logit link was used to explore the trajectory of psychological distress from 1 to 2 years after esophageal cancer surgery [23]. This model includes two random parameters, an intercept and a slope [24]. Detailed descriptions about this model are presented in the Supplementary Content 2. In addition, there were very few missing data in HADS and LOT-R, which were handled with mean imputation [25].

In order to assess whether dispositional optimism predicted the risk of psychological distress, we regressed the random intercept on the LOT-R sum score. Three hierarchical models were built to adjust for potential confounders. Model A was a crude model. Model B adjusted for sociodemographic covariates including age, sex, cohabitation status and education level, which are previously identified confounders. Model C further adjusted for clinical factors including comorbidity, neoadjuvant therapy, tumor stage, histology, and postoperative complication within 30 days post-surgery, which are potential but unidentified confounders. Details about the analysis process including model diagrams are presented in the Supplementary Content 2. In addition, we compared model B and model C using the likelihood ratio test.

Because the reliability analysis indicated that roughly half of the variation in the LOT-R sum score could be attributed to measurement error, and measurement error in the exposure can attenuate the regression coefficient [26], we conducted a sensitivity analysis using a latent (i.e., error free) factor to represent dispositional optimism. Three similar hierarchical models were built and related model diagrams are presented in Supplementary Content 2.

We used Mplus 8.2 (Muthén & Muthén, Los Angeles, USA) to build the latent growth curve models and Stata 13 (StataCorp, College Station, TX, USA) and SAS 9.4 (SAS Institute, Cary, NC, USA) for data cleaning. All 95% CIs were 2-sided.

## Results

### Characteristics of participants

Table 1 summarizes the characteristics of the 192 patients. The mean age was 66.3 years [standard deviation (SD), 8.5; range, 38.2 to 83.7], and 85.4% patients were male. The mean of LOT-R sum score was 15.2 (SD, 3.0; range, 6 to 20). There was no statistically significant difference in the LOT-R sum score between patients with different sociodemographic and clinical characteristics.

**Table 1:** Characteristics of the 192 patients with esophageal cancer surgery

	Number (%)
Age [mean (SD)]	66.3 (8.5)
Sex	
Female	28 (14.6)
Male	164 (85.4)
Cohabitation status	
Non-cohabitating	44 (22.9)
Cohabitating	148 (77.1)
Education level	
Nine-year compulsory school	48 (25.0)
Upper secondary school	85 (44.3)
Higher education	59 (30.7)
Tumor stage	
Complete regression after neoadjuvant therapy/I	71 (37.0)
II	62 (32.3)
III–IV	59 (30.7)
Tumor histology	
Adenocarcinoma	163 (84.9)
Squamous cell carcinoma	29 (15.1)
Postoperative complications (Clavien–Dindo grade)	
No complication	69 (35.9)
I–II	54 (28.1)
III–IV	69 (35.9)
Neoadjuvant therapy	
Yes	158 (82.3)
No	34 (17.7)
Surgical approach	
Total minimally invasive esophagectomy	52 (27.1)
Hybrid minimally invasive esophagectomy	63 (32.8)
Open esophagectomy	77 (40.1)
Charlson comorbidity index	
0	94 (49.0)
1	60 (31.3)
≥ 2	38 (19.8)
LOT sum score [mean (SD)]	15.2 (3.0)

*SD*, standard deviation; *LOT-R*, Life Orientation Test-Revised

### Trajectory of psychological distress from 1 to 2 years post-surgery

**Fig. 1:**
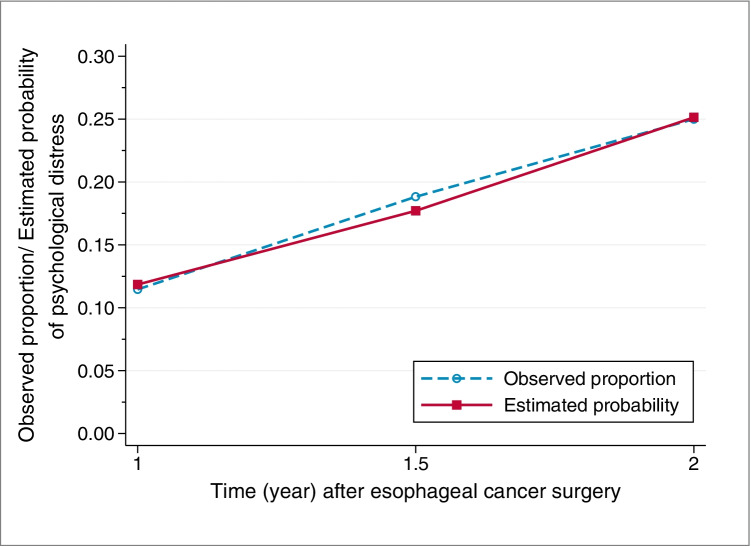
Observed proportion and estimated probability of clinically significant psychological distress after esophageal cancer surgery

One year after surgery, 11.5% (22 of 192) patients reported clinically significant psychological distress, and the proportion increased to 18.8% (32 of 170) at 1.5 years and to 25.0% (39 of 156) at 2 years post-surgery. The random intercept model with linear slope fit the data well, and the estimated probability of clinically significant psychological distress doubled from 1 year (11.8%) to 2 years (25.1%) after surgery, which is in line with the observed proportions (Fig. 1). In addition, there was no statistically significant variation in this longitudinal growth trajectory of psychological distress (p = 0.305), whereas substantial individual differences were found in the probability of reporting psychological distress at 1 year after surgery (variance = 23.17, p = 0.016).

### Predictive effect of dispositional optimism on psychological distress

**Fig. 2:**
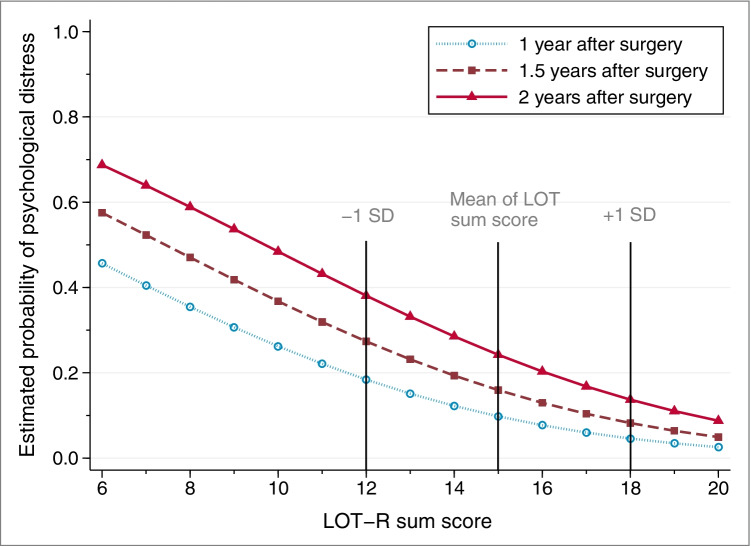
Estimated probability of psychological distress as a function of LOT-R sum score for three assessment time points after esophageal cancer surgery. Note. LOT-R: Life Orientation Test-Revised. The estimated probability is calculated from latent growth curve model, holding other included variables (age, sex, cohabitating status and education level) at their mean values

Higher dispositional optimism was associated with a lower risk of psychological distress at 1, 1.5, and 2 years after surgery and this association was not modified by time. Figure 2 displays the estimated probability of psychological distress as a function of LOT-R sum score, separated by different time points after surgery. In the crude model, for each point increase in the LOT-R sum score, the odds of reporting psychological distress decreased by 44% [odds ratio (OR) 0.56, 95% CI, 0.40 to 0.80; Table 2, model A]. The results remained almost unchanged after adjusting for sociodemographic covariates and clinical factors, with ORs equalling to 0.56 (95% CI, 0.40 to 0.79) and 0.55 (95% CI, 0.39 to 0.78), respectively (Table 2, model B and C). However, based on the likelihood ratio test, compared with the model B with adjustment for sociodemographic covariates, the model C with further adjustment for clinical factors did not show better model fit.

**Table 2:** Results from the three hierarchical random intercept models examining the predictive validity of dispositional optimism on psychological distress after esophageal cancer surgery

			Model A	Model B	Model C
Main analysis (LOT-R sum score)	Odds ratio	Unstandardized	0.56 (0.40, 0.80)	0.56 (0.40, 0.79)	0.55 (0.39, 0.78)
	*Log-odds*	*Fully standardized*	* − 0.36 (− 0.54, − 0.19)*	* − 0.37 (− 0.55, − 0.20)*	* − 0.38 (− 0.55, − 0.21)*
Sensitivity analysis (latent factor)	*Log-odds*	*Fully standardized*	* − 0.55 (− 0.65, − 0.44)*	* − 0.58 (− 0.81, − 0.36)*	* − 0.66 (− 0.90, − 0.42)*

Model A, crude mode; Model B, adjusted for age, sex, cohabitation status and education level; Model C, adjusted for age, sex, cohabitation status, education level, comorbidity, neoadjuvant therapy, tumor stage, histology, and postoperative complication within 30 days post-surgery; LOT-R, Life Orientation Test-Revised. Main analysis, models used LOT-R sum score to represent dispositional optimism; Sensitivity analysis, models incorporated factor analysis and used latent factor to represent dispositional optimism. Full standardization means both the independent and the dependent variables were standardized

Sensitivity analysis fitting a latent factor to represent dispositional optimism also demonstrated similar results among the three hierarchical models, with fully standardized log-odds equalling to − 0.55, − 0.58, and − 0.66, respectively (Table 2, sensitivity analysis). However, the coefficients from the sensitivity analysis were larger than the coefficients from the main analysis. For example, in the crude model, with 1 SD increase in the dispositional optimism, the log-odds decreased 0.36 SD in the main analysis, whereas the log-odds decreased 0.55 SD in the sensitivity analysis (Table 2, model A). This difference is most likely because measurement error in the exposure could attenuate the observed coefficient [26], and the sensitivity analysis using latent factor has removed the measured error in the LOT-R.

## Discussion

This Swedish nationwide population-based longitudinal study showed that the proportion of self-reported clinically significant psychological distress roughly doubled from 1 to 2 years after esophageal cancer surgery. Moreover, higher dispositional optimism predicted a lower risk of psychological distress at 1, 1.5, and 2 years post-surgery.

The self-reported psychological distress at 1 year after esophageal cancer surgery was 11.5% in the current study, which was lower than the proportions reported by one previous study conducted in the UK [5]. The inconsistency might be because the previous study was only conducted in one British hospital while the current study was Swedish nationwide population–based. Moreover, another previous study with large sample size using British hospital and primary care databases has found that 13.9% patients had psychiatry morbidity based on the diagnosis and prescription codes within 1 year after esophageal cancer surgery [27], which is similar to the proportion found in the current study. In addition, given that non-participation in the current study was mainly related to unwillingness, severe illness and cancer recurrence [16], the non-participants might suffer more from psychological distress compared with the participants, which could make the observed proportion underestimated.

The current study found that the proportion of psychological distress increased from 1 to 2 years after esophageal cancer surgery. This indicates unmet demands for early psychological screening and timely interventions to improve mental health. After esophageal cancer surgery, patients often face lingering symptoms (e.g., dumping, reflux and eating difficulty), malnutrition, decreased HRQL and risk of cancer recurrence [3, 28]. Moreover, most patients with esophageal cancer are old [29, 30], and may have comorbidities relating to aging and risk factors associated with cancer [29]. These chronic stresses could induce increased glucocorticoid stress hormones, cause structural change in the brain, and result in persistent vulnerability to mental illness [31], therefore leading to increased psychological distress over time after esophageal cancer surgery. Interventions helping patients cope with these postoperative challenges may contribute to the improvement of mental health.

The present study further found that higher dispositional optimism predicted a lower probability of self-reported psychological distress after esophageal cancer surgery. This result is in line with previous studies [10–14]. Potential mechanisms for the protective effect of dispositional optimism may be related to coping strategies, goal adjustment and social support [32–37]. Studies have shown that more optimistic people seem to be more flexible in coping and goal adjustment. When the situation is perceived as controllable, more optimistic people tend to persistently seek measures to overcome the adversity, but if the situation is perceived as uncontrollable, they are also more likely to accept the reality and disengage from the unattainable goals rapidly [32–35]. These strategies may lead to less negative experiences and rumination and therefore better mental health [32–35]. In addition, people with higher dispositional optimism are prone to receive more social support due to their positive outlook [36, 37], which may help relieve the perceived stress and thus reduce the risk of psychological distress.

The predictive effect of dispositional optimism has both clinical and research implications. Dispositional optimism may not only be used to predict prognosis, but also help differentiate patients with similar sociodemographic and clinical characteristics to identify those needing additional psychological support. Early identification of high-risk patients could lead to more frequent mental health surveillance and timely psychological interventions, thereby ameliorating or perhaps even preventing later psychological distress. Moreover, compared with general surveillance, such a risk-based strategy may be more cost-effective and could allocate medical resources to patients with more dire needs. In addition, because dispositional optimism appears at least partly modifiable through psychological interventions [15], it would be worth investigating whether psychological distress after esophageal cancer surgery could be prevented or treated through increasing dispositional optimism. Results from the present study provide a base for future interventional studies.

This study has several methodological strengths. First, it is the first longitudinal study examining the association between dispositional optimism and psychological distress among patients with esophageal cancer. Second, the prospective, nationwide, and longitudinal design facilitates its generalizability and enables the assessment of trajectory over time. Third, we conducted a series of confirmatory factor analysis to determine the proper factor structure of LOT-R, and to the best of our knowledge, it is the first study assessing the dimensionality of LOT-R among patients with esophageal cancer. Moreover, in addition to the observed LOT-R sum score, we used a latent factor to represent dispositional optimism, which further confirmed the predictive validity of dispositional optimism by removing the potential bias attributable to measurement error in the LOT-R.

This study also has limitations. First, psychological distress was measured by a self-reported screening questionnaire HADS instead of being defined by clinical diagnosis. Although HADS has been recommended to be used in oncology settings [38, 39], such subjective reporting might result in underestimation [40]. Second, less optimistic patients and those suffering from psychological distress might be prone to decline the participation, withdraw or miss the follow-ups, which could make the observed proportion of psychological distress and the protective effect of dispositional optimism underestimated. Third, this observational study found that dispositional optimism predicted postoperative psychological distress, but prediction does not mean causality. Unmeasured common causes such as genetic factors [41] could result in a spurious association between dispositional optimism and psychological distress, and whether increasing dispositional optimism could ameliorate psychological distress needs to be examined by future interventional studies.

In conclusion, this study found that psychological distress increased over time from 1 to 2 years after esophageal cancer surgery and higher dispositional optimism predicted a lower risk of psychological distress. It highlights the need for timely psychological screening and interventions. In addition, early assessment of dispositional optimism may help identify patients with higher risk of developing psychological distress after esophageal cancer surgery, thereby providing personalized mental health surveillances and tailored psychological interventions, and contributing to the prevention of postoperative psychological distress.

## Supplementary Information

Below is the link to the electronic supplementary material.

Supplementary file1 (PDF 1327 KB)

## Data Availability

The data that support the findings of this study are available on request from the corresponding author. The data are not publicly available due to privacy or ethical restrictions.

## Code availability

Codes supporting the findings of this study are stored at the server of Karolinska Institutet, and they can be made available upon request.

## References

[1] Sung H, Ferlay J, Siegel RL, Laversanne M, Soerjomataram I, Jemal A, Bray F (2021). Global cancer statistics 2020: GLOBOCAN estimates of incidence and mortality worldwide for 36 cancers in 185 countries. CA Cancer J Clin.

[2] Office for National Statistics (2019) Cancer survival in England - adults diagnosed. https://www.ons.gov.uk/peoplepopulationandcommunity/healthandsocialcare/conditionsanddiseases/datasets/cancersurvivalratescancersurvivalinenglandadultsdiagnosed. Accessed 27 Jun 2021

[3] Lagergren J, Smyth E, Cunningham D, Lagergren P (2017). Oesophageal cancer. Lancet.

[4] Johansson R, Carlbring P, Heedman A, Paxling B, Andersson G (2013). Depression, anxiety and their comorbidity in the Swedish general population: point prevalence and the effect on health-related quality of life. PeerJ.

[5] Hellstadius Y, Lagergren J, Zylstra J, Gossage J, Davies A, Hultman CM, Lagergren P, Wikman A (2017). A longitudinal assessment of psychological distress after oesophageal cancer surgery. Acta Oncol.

[6] Hellstadius Y, Lagergren J, Zylstra J, Gossage J, Davies A, Hultman CM, Lagergren P, Wikman A (2016). Prevalence and predictors of anxiety and depression among esophageal cancer patients prior to surgery. Dis Esophagus.

[7] Linden W, Vodermaier A, Mackenzie R, Greig D (2012). Anxiety and depression after cancer diagnosis: prevalence rates by cancer type, gender, and age. J Affect Disord.

[8] Hellstadius Y, Lagergren P, Lagergren J, Johar A, Hultman CM, Wikman A (2015). Aspects of emotional functioning following oesophageal cancer surgery in a population-based cohort study. Psychooncology.

[9] Carver CS, Scheier MF, Segerstrom SC (2010). Optimism. Clin Psychol Rev.

[10] Ai AL, Carretta H (2020). Optimism/hope associated with low anxiety in patients with advanced heart disease controlling for standardized cardiac confounders. J Health Psychol.

[11] David D, Montgomery GH, Bovbjerg DH (2006). Relations between coping responses and optimism-pessimism in predicting anticipatory psychological distress in surgical breast cancer patients. Pers Individ Dif.

[12] Zenger M, Brix C, Borowski J, Stolzenburg JU, Hinz A (2010). The impact of optimism on anxiety, depression and quality of life in urogenital cancer patients. Psychooncology.

[13] Horney DJ, Smith HE, McGurk M, Weinman J, Herold J, Altman K, Llewellyn CD (2011). Associations between quality of life, coping styles, optimism, and anxiety and depression in pretreatment patients with head and neck cancer. Head Neck.

[14] Rajandram RK, Ho SM, Samman N, Chan N, McGrath C, Zwahlen RA (2011). Interaction of hope and optimism with anxiety and depression in a specific group of cancer survivors: a preliminary study. BMC Res Notes.

[15] Malouff JM, Schutte NS (2016). Can psychological interventions increase optimism? A meta-analysis. J Posit Psychol.

[16] Schandl A, Johar A, Anandavadivelan P, Vikstrom K, Malberg K, Lagergren P (2020). Patient-reported outcomes 1 year after oesophageal cancer surgery. Acta Oncol.

[17] Liu Y, Pettersson E, Schandl A, Markar S, Johar A, Lagergren P (2021). Higher dispositional optimism predicts better health-related quality of life after esophageal cancer surgery: a nationwide population-based longitudinal study. Ann Surg Oncol.

[18] Zigmond AS, Snaith RP (1983). The hospital anxiety and depression scale. Acta Psychiatr Scand.

[19] Bjelland I, Dahl AA, Haug TT, Neckelmann D (2002). The validity of the Hospital Anxiety and Depression Scale. An updated literature review. J Psychosom Res.

[20] Hranov LG (2007). Comorbid anxiety and depression: illumination of a controversy. Int J Psychiatry Clin Pract.

[21] Muhonen T, Torkelson EVA (2013). Kortversioner av frågeformulär inom arbets- och hälsopsykologi—om att mäta coping och optimism. Nordisk Psykologi.

[22] Scheier MF, Carver CS, Bridges MW (1994). Distinguishing optimism from neuroticism (and trait anxiety, self-mastery, and self-esteem): a reevaluation of the Life Orientation Test. J Pers Soc Psychol.

[23] Lee TK, Wickrama K, O’Neal CW (2018). Application of latent growth curve analysis with categorical responses in social behavioral research. Struct Equ Modeling.

[24] Duncan TE, Duncan SC (2009). The ABC’s of LGM: an introductory guide to latent variable growth curve modeling. Soc Personal Psychol Compass.

[25] Bell ML, Fairclough DL, Fiero MH, Butow PN (2016). Handling missing items in the Hospital Anxiety and Depression Scale (HADS): a simulation study. BMC Res Notes.

[26] Hutcheon JA, Chiolero A, Hanley JA (2010). Random measurement error and regression dilution bias. BMJ.

[27] Bouras G, Markar SR, Burns EM, Mackenzie HA, Bottle A, Athanasiou T, Hanna GB, Darzi A (2016). Linked hospital and primary care database analysis of the incidence and impact of psychiatric morbidity following gastrointestinal cancer surgery in England. Ann Surg.

[28] Pinto E, Cavallin F, Scarpa M (2019). Psychological support of esophageal cancer patient?. J Thorac Dis.

[29] Smyth EC, Lagergren J, Fitzgerald RC, Lordick F, Shah MA, Lagergren P, Cunningham D (2017). Oesophageal cancer. Nat Rev Dis Primers.

[30] Cancer Research UK (2021) Oesophageal cancer statistics. https://www.cancerresearchuk.org/health-professional/cancer-statistics/statistics-by-cancer-type/oesophageal-cancer#heading-Zero. Accessed 29 July 2021

[31] Chetty S, Friedman AR, Taravosh-Lahn K, Kirby ED, Mirescu C, Guo F, Krupik D, Nicholas A, Geraghty A, Krishnamurthy A, Tsai MK, Covarrubias D, Wong A, Francis D, Sapolsky RM, Palmer TD, Pleasure D, Kaufer D (2014). Stress and glucocorticoids promote oligodendrogenesis in the adult hippocampus. Mol Psychiatry.

[32] Nes LS, Segerstrom SC (2006). Dispositional optimism and coping: a meta-analytic review. Pers Soc Psychol Rev.

[33] Buyukasik-Colak C, Gundogdu-Akturk E, Bozo O (2012). Mediating role of coping in the dispositional optimism-posttraumatic growth relation in breast cancer patients. J Psychol.

[34] Ramirez-Maestre C, Esteve R, Lopez-Martinez AE, Serrano-Ibanez ER, Ruiz-Parraga GT, Peters M (2019). Goal adjustment and well-being: the role of optimism in patients with chronic pain. Ann Behav Med.

[35] Willis K, Timmons L, Pruitt M, Schneider HL, Alessandri M, Ekas NV (2016). The relationship between optimism, coping, and depressive symptoms in hispanic mothers and fathers of children with autism spectrum disorder. J Autism Dev Disord.

[36] Trunzo JJ, Pinto BM (2003). Social support as a mediator of optimism and distress in breast cancer survivors. J Consult Clin Psychol.

[37] Garner MJ, McGregor BA, Murphy KM, Koenig AL, Dolan ED, Albano D (2015). Optimism and depression: a new look at social support as a mediator among women at risk for breast cancer. Psychooncology.

[38] Castelli L, Binaschi L, Caldera P, Mussa A, Torta R (2011). Fast screening of depression in cancer patients: the effectiveness of the HADS. Eur J Cancer Care (Engl).

[39] Annunziata MA, Muzzatti B, Altoe G (2011). Defining hospital anxiety and depression scale (HADS) structure by confirmatory factor analysis: a contribution to validation for oncological settings. Ann Oncol.

[40] Thalen-Lindstrom AM, Glimelius BG, Johansson BB (2016). Identification of distress in oncology patients: a comparison of the Hospital Anxiety and Depression Scale and a Thorough Clinical Assessment. Cancer Nurs.

[41] Mosing MA, Zietsch BP, Shekar SN, Wright MJ, Martin NG (2009). Genetic and environmental influences on optimism and its relationship to mental and self-rated health: a study of aging twins. Behav Genet.

