# Supplementary material for: Psychological distress after esophageal cancer surgery and the predictive effect of dispositional optimism: a nationwide population-based longitudinal study
Source: Support Care Cancer. 2021 Sep 4;30(2):1315–22. doi: 10.1007/s00520-021-06517-x (PMC8727404; doi:10.1007/s00520-021-06517-x)
Supplement: Supplementary file 1 — Supplementary file1 (PDF 1327 KB) [file 520_2021_6517_MOESM1_ESM.pdf]

Supplementary information

Journal: Supportive care in cancer

Article Title

Psychological distress after esophageal cancer surgery and the predictive effect of dispositional optimism: a nationwide population-based longitudinal study

Authors

Yangjun Liu, MD; Erik Pettersson, PhD; Anna Schandl, PhD; Sheraz Markar, PhD; Asif Johar, Msc; Pernilla Lagergren, PhD

Corresponding Author

Pernilla Lagergren, Department of Molecular Medicine and Surgery, Karolinska Institutet

E-mail: pernilla.lagergren@ki.se

Supplementary Content

Supplementary Content 1: Confirmatory factor analysis for Life Orientation Test-Revised (LOT-R)………………………………………………………………… Page 2 - 17

Supplementary Content 2: Latent growth curve model……………………… Page 18 - 23

Supplementary Content 1

Confirmatory factor analysis for Life Orientation Test-Revised (LOT-R)

Introduction

Dispositional optimism is commonly measured with the Life Orientation Test-Revised (LOT-R), which consists of three positively worded items, three negatively worded items, and four filler items [1]. It is conceptualized as a continuous measure [2], and mostly treated as a bipolar unidimensional scale, with optimism and pessimism at the opposite end of a single spectrum [1,3]. However, some studies have also suggested that LOT-R is a bi-dimensional scale, with two partially independent factors, namely, optimism and pessimism [4-7]. Nevertheless, some researchers have argued that the two-factor structure might be attributed to a methodological artefact [8-10], and proposed a revised unidimensional structure, where one general factor represents dispositional optimism, on which all items load, and one method factor includes the positively (or negatively) worded items [8-11].

Given the lack of consensus about the dimensionality of the LOT-R, and that no study to date has assessed the psychometric property of LOT-R among patients with esophageal cancer, we conducted confirmatory factor analysis (CFA) for LOT-R based on our Swedish nationwide population-based data.

Methods

Participants

Data for the present analysis is from a prospective, ongoing Swedish-nationwide cohort study entitled Oesophageal Surgery on Cancer patients - Adaptation and Recovery (OSCAR). Detailed description of the OSCAR study can be found elsewhere [12]. In total, 197 patients

who underwent esophageal cancer surgery between January 1, 2013 and December 31, 2016 and answered the LOT-R were included in the analysis.

Swedish version of LOT-R

The Swedish version of LOT-R scale has been validated, and consists of six items (the four filler items were removed) [13]. Patients were asked to report their agreement with each 5-point Likert item, ranging from 0 ("strongly disagree") to 4 ("strongly agree") [1].

Statistical Analysis

We conducted descriptive analysis and examined the distribution of response to each item. Additionally, we calculated the correlation between all items. CFA with robust maximum likelihood estimation (MLR) was used to assess the dimensionality of LOT-R. We evaluated the model fit of four potential models reported in previous studies (sFigure 1). Model 1 was a congeneric one-factor model positing one dispositional optimism factor without any correlated uniqueness. Model 2 was an oblique two-factor model presuming optimism and pessimism are two correlated factors. Model 3 assumed one general factor (dispositional optimism) plus a method factor associated with positively worded item, while model 4 contained one general factor (dispositional optimism) plus a method factor associated with reversed negatively worded items. Model fit was assessed by $\chi 2$ test, root mean square error of approximation (RMSEA), standardized root mean square residual (SRMR), comparative fit index (CFI), and Akaike's information criterion (AIC). RMSEA and SRMR are absolute indices, with RMSEA value < 0.05 and SRMR value < 0.08 indicating good overall model fit [14]. CFI compares the target model with the baseline model, and value > 0.95 represents good model fit [14]. AIC evaluates both goodness of fit and parsimony, and the model with lower AIC value is preferred [15]. Nested models were compared by the Satorra-Bentler scaling correction likelihood ratio ($\Delta\chi 2$) test [16].

In addition, we estimated internal reliability, which is the variance in the observed measure that can be attributed to a corresponding and unobserved latent construct. We adopted McDonald's omega (ω) as the indicator, because it is based on the actual measurement model and allows items to load freely on factors [17]. Omega total (ω_t) is the variance ratio explained by both the general factor and the method factor, whereas omega hierarchical (ω_h) is the variance ratio explained only by the general factor [17]. We calculated ω_t for all models and ω_h for the models with method factor. We used bootstrap to compute the 95% confidence intervals (CIs) for ω. All analyses were performed using Mplus version 8.2 (Muthén & Muthén, Los Angeles, USA).

Results

Patients' response to each item

Descriptive statistics of patients' responses to each item are displayed in sTable 1. Among the included 197 patients, 13 (7%) patients responded to part of the items, and most missing (8 patients, 4%) occurred in the first negatively worded item (item N2, "if something can go wrong for me, it will"). Furthermore, compared with other items, response to item N2 had unusually large variation. sFigure 2 displays the distribution of patients' response to each item. All responses were skewed to one direction (more positive or less negative) except for item N2, which was bimodal. Moreover, item N2 had positive correlation with both positively and negatively worded items (sTable 2).

Model fit and omega value for potential models of LOT-R with six items

The fit indices for the assessed four potential models are presented in sTable 3. The model 4 assuming one general factor (dispositional optimism) plus a method factor associated with reversed negatively worded items had the lowest AIC, and fit the data best, with Yuan-Bentler $\chi 2$ (6) = 8.158 ($p = 0.227$), CFI = 0.983, RMSEA = 0.043 and SRMR = 0.039. These results

indicated that LOT-R was unidimensional, but with an artificial method factor associated with reversed negatively worded items. However, the loading of item N2 in this model was negative even though the score has already been reversed to account for the negative wording (sTable 4). In addition, McDonald's omega values for the four models were not satisfying (range: ω_t 0.494 to 0.578, ω_h: 0.285 to 0.492). In model 4, which showed the best fit, the ratio of total variance explained by general factor (dispositional optimism) was 0.285, with 95% bootstrapped CI from 0.108 to 0.427 (sTable 3).

Removal of the first negatively worded item (item N2)

The above results showed that patients' response to the first negatively worded item (item N2) in LOT-R was bimodal, and it had equivocal correlation with both positively worded items and negatively worded items. Moreover, its loading was negative in the best-fit model even though it has already been reversed to account for the negative wording. Because it is the first negatively worded item in the LOT-R scale, patients might misunderstand the wording and scoring method, which could cause the bimodal distribution of the response and ensuing large variation and negative loading. In addition, patients filled in the LOT-R during an on-site interview, and they could check with the research nurse if they found any item unclear. However, only few patients expressed confusion about the LOT-R and they did not express specific confusion about the first negatively worded item. Furthermore, this item had the largest percentage of missingness, and patient representatives in our research partnership group reported that the hypothetical wording ("If something can go wrong for me, it will") reminded cancer recurrence to some extent. Thus, there might be another possibility that patients were confused on this item but they were reluctant to check with the research nurse due to accompanying uncomfortable feeling or social desirability, or patients answered this item not based on their generalized expectations for future outcomes but specific worries about cancer recurrence or death. In order to remove the noise caused by this item, we

excluded it and reassessed the model fit of four similar models based on the remaining five items (model diagrams were presented in sFigure 3).

Model fit and omega value for potential models of LOT-R with five items

The model fit for the four rebuilt models are displayed in sTable 5. The model 3 with one general factor (dispositional optimism) plus a method factor associated with positively worded items showed poor RMSEA (0.111) and the null hypothesis that the model fit the data was rejected (Yuan-Bentler $\chi2$ (2) = 6.858, p = 0.032), even though this model had good CFI (0.953) and SRMR (0.018) values, and relatively low AIC (2735.815). The other two models, oblique two-factor model (model 2) and one general factor (dispositional optimism) model with correlated errors between reversed negatively worded items (model 4) showed the same best model fit (Yuan-Bentler $\chi2$ (4) = 5.854, p = 0.210; AIC = 2734.293; CFI = 0.982; RMSEA = 0.049; SRMR = 0.035). After removing the first negatively worded item, McDonald's omega values for all reassessed models were increased (range: ω_t 0.568 – 0.724, ω_h: 0.486 – 0.496).

Because the one-dimensional bi-polar model has a stronger theoretical base [2], we chose the one general factor (dispositional optimism) model with correlated errors between reversed negatively worded items (model 4) in the present study, and the total variance explained by the general factor (dispositional optimism) was 0.486 (95% bootstrapped CI: 0.306 to 0.616).

References

1. Scheier MF, Carver CS, Bridges MW (1994) Distinguishing optimism from neuroticism (and trait anxiety, self-mastery, and self-esteem): a reevaluation of the Life Orientation Test. J Pers Soc Psychol 67 (6):1063-1078. doi:10.1037//0022-3514.67.6.1063

2. Carver CS, Scheier MF, Segerstrom SC (2010) Optimism. Clinical psychology review 30 (7):879-889. doi:10.1016/j.cpr.2010.01.006

3. Mehrabian A, Ljunggren E (1997) Dimensionality and content of optimism-pessimism analyzed in terms of the pad temperament model. Personality and Individual Differences 23 (5):729-737. doi:10.1016/s0191-8869(97)00119-0

4. Herzberg PY, Glaesmer H, Hoyer J (2006) Separating optimism and pessimism: a robust psychometric analysis of the revised Life Orientation Test (LOT-R). Psychol Assess 18 (4):433-438. doi:10.1037/1040-3590.18.4.433

5. Glaesmer H, Rief W, Martin A, Mewes R, Brahler E, Zenger M, Hinz A (2012) Psychometric properties and population-based norms of the Life Orientation Test Revised (LOT-R). British journal of health psychology 17 (2):432-445. doi:10.1111/j.2044-8287.2011.02046.x

6. Zenger M, Finck C, Zanon C, Jimenez W, Singer S, Hinz A (2013) Evaluation of the Latin American version of the Life Orientation Test-Revised. Int J Clin Hlth Psyc 13 (3):243-252. doi:10.1016/s1697-2600(13)70029-2

7. Creed PA, Patton W, Bartrum D (2002) Multidimensional Properties of the Lot-R: Effects of Optimism and Pessimism on Career and Well-Being Related Variables in Adolescents. Journal of Career Assessment 10 (1):42-61. doi:10.1177/1069072702010001003

8. Rauch WA, Schweizer K, Moosbrugger H (2007) Method effects due to social desirability as a parsimonious explanation of the deviation from unidimensionality in LOT-R scores. Personality and Individual Differences 42 (8):1597-1607. doi:10.1016/j.paid.2006.10.035

9. Vautier S, Raufaste E, Cariou M (2003) Dimensionality of the Revised Life Orientation Test and the status of filler items. International Journal of Psychology 38 (6):390-400. doi:10.1080/00207590344000222

10. Vecchione M, Alessandri G, Caprara GV, Tisak J (2014) Are Method Effects Permanent or Ephemeral in Nature? The Case of the Revised Life Orientation Test. Structural Equation Modeling: A Multidisciplinary Journal 21 (1):117-130. doi:10.1080/10705511.2014.859511

11. Pan TM, Mills SD, Fox RS, Baik SH, Harry KM, Roesch SC, Sadler GR, Malcarne VL (2017) The Psychometric Properties of English and Spanish Versions of the Life Orientation Test-Revised in Hispanic Americans. J Psychopathol Behav Assess 39 (4):657-668. doi:10.1007/s10862-017-9606-x

12. Schandl A, Johar A, Anandavadivelan P, Vikstrom K, Malberg K, Lagergren P (2020) Patient-reported outcomes 1 year after oesophageal cancer surgery. Acta Oncol 59 (6):613-619. doi:10.1080/0284186X.2020.1741677

13. Muhonen T, Torkelson EVA (2005) Kortversioner av frågeformulär inom arbets- och hälsopsykologi—om att mäta coping och optimism. Nordisk Psykologi 57 (3):288-297. doi:10.1080/00291463.2005.10637375

14. Hu Lt, Bentler PM (1999) Cutoff criteria for fit indexes in covariance structure analysis: Conventional criteria versus new alternatives. Structural Equation Modeling: A Multidisciplinary Journal 6 (1):1-55. doi:10.1080/10705519909540118

15. Vandekerckhove J, Matzke D, Wagenmakers E-J (2015) Model comparison and the principle of parsimony. In: The Oxford handbook of computational and mathematical psychology. Oxford library of psychology. Oxford University Press, New York, NY, US, pp 300-319

16. Satorra A, Bentler PM (2010) Ensuring Positiveness of the Scaled Difference Chi-square Test Statistic. Psychometrika 75 (2):243-248. doi:10.1007/s11336-009-9135-y

17. Revelle W, Condon DM (2019) Reliability from alpha to omega: A tutorial. Psychol Assess 31 (12):1395-1411. doi:10.1037/pas0000754

sTable 1. Descriptive statistics for the six items of Life Orientation Test-Revised answered by 197 patients with esophagectomy for cancer

Item	Sample size	Mean	Variance	Skewness	Kurtosis
P1	191	3.052	0.992	-0.836	0.149
P3	197	3.269	0.846	-1.143	0.715
P6	192	3.115	1.174	-1.358	1.268
N2	189	2.053	2.241	-0.128	-1.455
N4	193	1.358	1.307	0.539	-0.557
N5	194	1.113	1.265	0.819	-0.198

Note. P1, P2, and P3 represent positively worded items 1, 3, and 6, respectively. N2, N4 and N5 represent negatively worded items 2, 4 and 5, respectively.

sTable 2. Item intercorrelations in Life Orientation Test-Revised

	P1	P3	P6	N2	N4	N5
P1	1.000					
P3	0.400	1.000				
P6	0.300	0.303	1.000			
**N2**	**0.209**	**0.105**	**0.014**	**1.000**		
N4	-0.089	-0.074	-0.218	**0.232**	1.000	
N5	-0.147	-0.207	-0.223	**0.234**	0.548	1.000

Note. P1, P2, and P3 represent positively worded items 1, 3, and 6, respectively. N2, N4 and N5 represent negatively worded items 2, 4 and 5, respectively.

sTable 3. Fit statistics and McDonald's omega for the assessed four potential structures of Life Orientation Test-Revised (six items)

Model (6 items)	Y–B $\chi2$ (*df*)	*p*	AIC	CFI	RMSEA	SRMR	$\Delta\chi2_{S-B}$ (*df*)	*p*	McDonald's omega (95% bootstrapped CI)
1. Congeneric one-factor model									
	72.219 (9)	< 0.001	3459.439	0.510	0.189	0.114	Reference model		ω_t: 0.578 (0.445, 0.665)
2. Oblique two-factor model									
	24.799 (8)	0.002	3417.770	0.870	0.103	0.069	40.400 (1)	< 0.001	ω_t: 0.494 (0.313, 0.607)
3. One general factor (dispositional optimism) plus a method factor associated with positively worded items									
	24.929 (6)	< 0.001	3418.561	0.853	0.127	0.063	38.447 (3)	< 0.001	ω_t: 0.664 (0.570, 0.720)<br>ω_h: 0.492 (0.378, 0.583)
4. One general factor (dispositional optimism) plus a method factor associated with reversed negatively worded items									
	8.158 (6)	0.227	3408.122	0.983	0.043	0.039	85.001 (3)	< 0.001	ω_t: 0.640 (0.532, 0.712)<br>ω_h: 0.285 (0.108, 0.427)

Note. Y–B: Yuan-Bentler; df: degrees of freedom; p: p value; AIC: Akaike information criterion; CFI: comparative fit index; RMSEA: root mean square error of approximation; SRMR: standardized root mean square residual; CI: confidence interval; ω_t: McDonald's omega total; ω_h: McDonald's omega hierarchical.

sTable 4. Loadings of the six items of Life Orientation Test-Revised in the model assuming one general factor (dispositional optimism) plus a method factor associated with reversed negatively worded items

Item	Unstandardized factor loading	Standardized factor loading
**General factor (dispositional optimism)**		
P1: In uncertain times, I usually expect the best.		
	1.000	0.644
P3: I am always optimistic about my future.		
	0.881	0.567
P6: Overall, I expect more good things to happen to me than bad.		
	0.814	0.524
re_N2: If something can go wrong for me, it will.		
	**-0.503**	**-0.324**
re_N4: I hardly ever expect things to go my way.		
	0.337	0.217
re_N5: I rarely count on good things happening to me.		
	0.538	0.346
**Method factor**		
re_N2: If something can go wrong for me, it will.		
	1.000	0.612
re_N4: I hardly ever expect things to go my way.		
	1.250	0.765
re_N5: I rarely count on good things happening to me.		
	1.352	0.828

Note. P1, P2, and P3 represent positively worded items 1, 3, and 6, respectively. N2, N4 and N5 represent negatively worded items 2, 4 and 5, respectively. re_N2, re_N4 and re_N5 represent reversed negatively worded items 2, 4 and 5, respectively.

sTable 5. Fit statistics and McDonald's omega for the reassessed four potential structures of Life Orientation Test-Revised (five items)

Model (5 items)	Y–B $\chi2$ (*df*)	*p*	AIC	CFI	RMSEA	SRMR	$\Delta\chi2_{S-B}$ (*df*)	*p*	McDonald's omega (95% bootstrapped CI)
1. Congeneric one-factor model									
	57.139 (5)	< 0.001	2773.929	0.492	0.230	0.104	Reference model		ω_t: 0.622 (0.483, 0.718)
2. Oblique two-factor model									
	5.853 (4)	0.210	2734.293	0.982	0.048	0.035	190.216 (1)	< 0.001	ω_t: 0.568 (0.361, 0.794)
3. One general factor (dispositional optimism) plus a method factor associated with positively worded items									
	6.858 (2)	0.032	2735.815	0.953	0.111	0.018	41.450 (3)	< 0.001	ω_t: 0.724 (0.568, 0.780) ω_h: 0.496 (0.349, 0.593)
4. One general factor (dispositional optimism) model with correlated errors between reversed negatively worded items									
	5.854 (4)	0.210	2734.293	0.982	0.049	0.035	189.867 (3)	< 0.001	ω_h: 0.486 (0.306, 0.616)

Note. Y–B: Yuan-Bentler; df: degrees of freedom; p: p value; AIC: Akaike information criterion; CFI: comparative fit index; RMSEA: root mean square error of approximation; SRMR: standardized root mean square residual; CI: confidence interval; ω_t: McDonald's omega total; ω_h: McDonald's omega hierarchical.

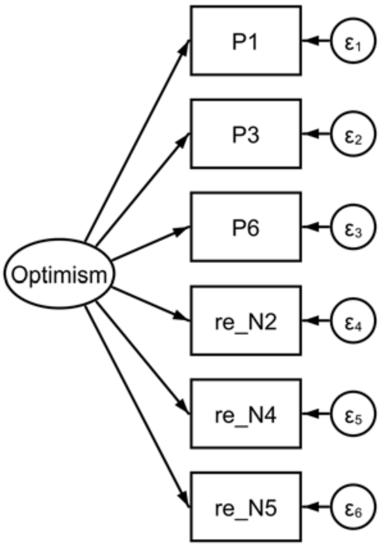

Model 1. Congeneric one-factor model

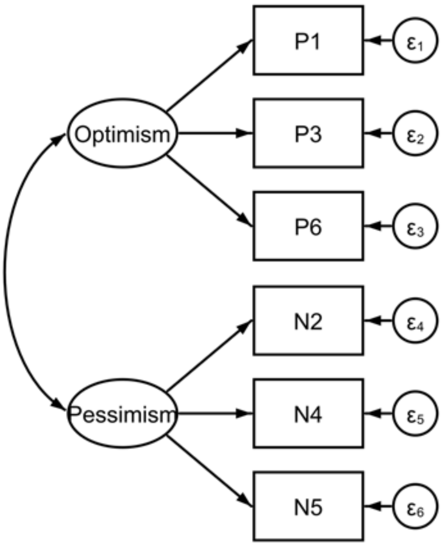

Model 2. Oblique two-factor model

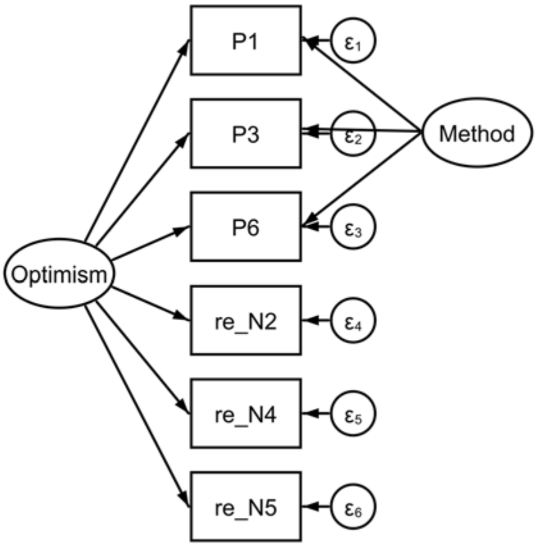

Model 3. One general factor (dispositional optimism) plus a method factor associated with positively worded items

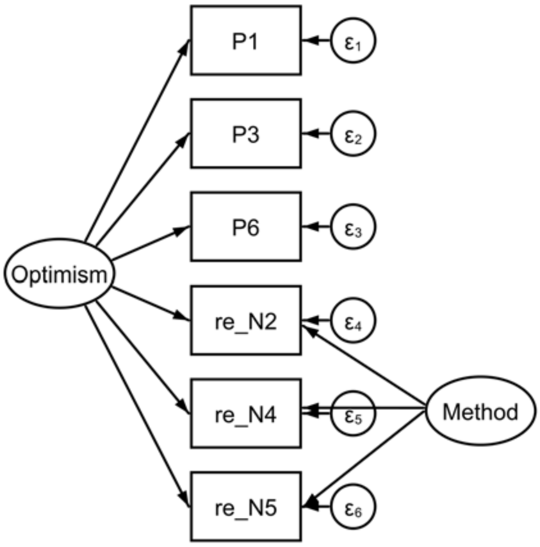

Model 4. One general factor (dispositional optimism) plus a method factor associated with reversed negatively worded items

sFigure 1. Assessed models for Life Orientation Test-Revised (six items)

Note. P1, P2, and P3 represent positively worded items 1, 3, and 6, respectively. N2, N4 and N5 represent negatively worded items 2, 4 and 5, respectively. re_N2, re_N4 and re_N5 represent reversed negatively worded items 2, 4 and 5, respectively.

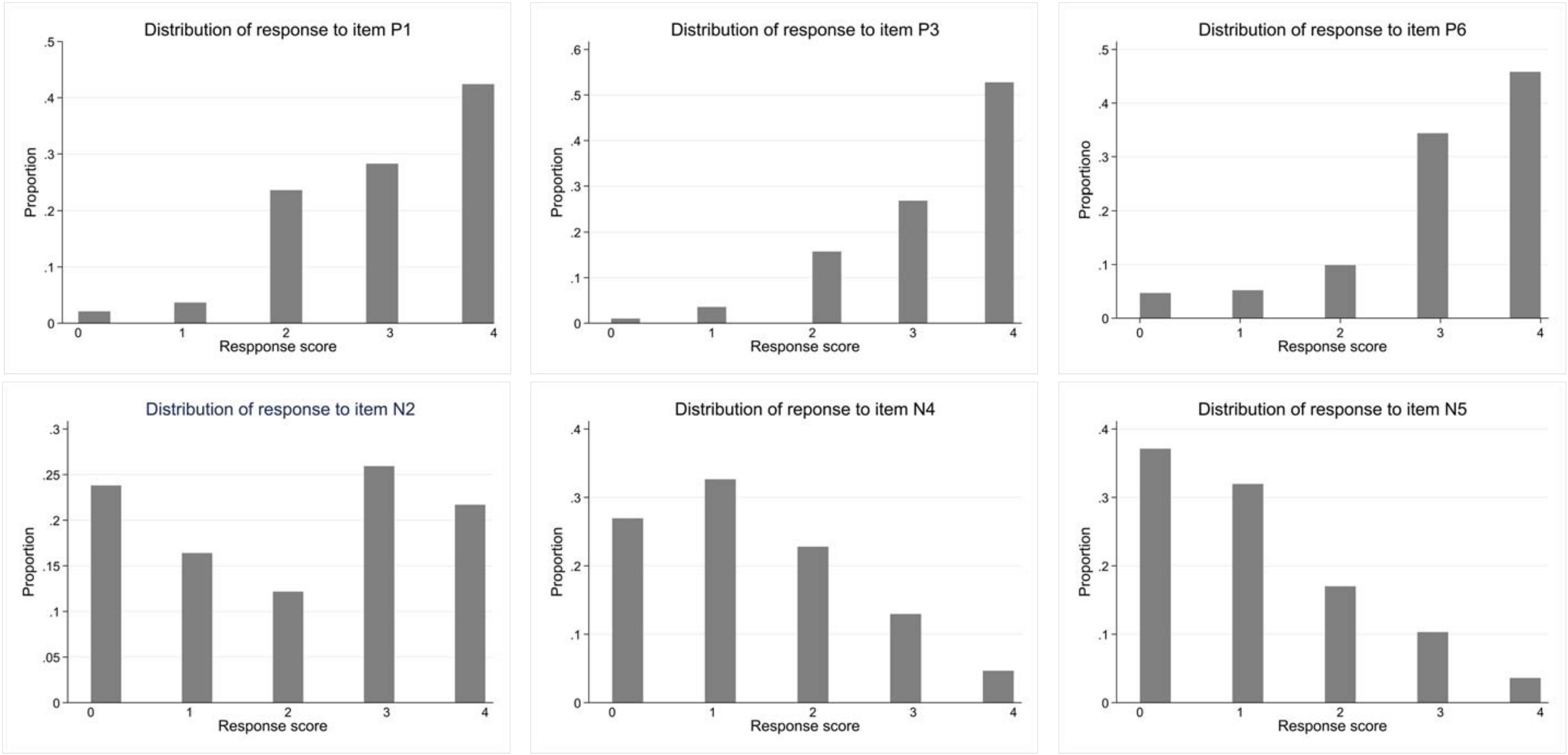

sFigure 2. Distribution of response to each item in Life Orientation Test-Revised

Note. P1, P2, and P3 represent positively worded items 1, 3, and 6, respectively. N2, N4 and N5 represent negatively worded items 2, 4 and 5, respectively. Score 0: “I disagree a lot”; score 1: “I disagree a little”’ score 2: “I neither agree nor disagree”; score 3: “I agree a little”; score 4: “I agree a lot”.

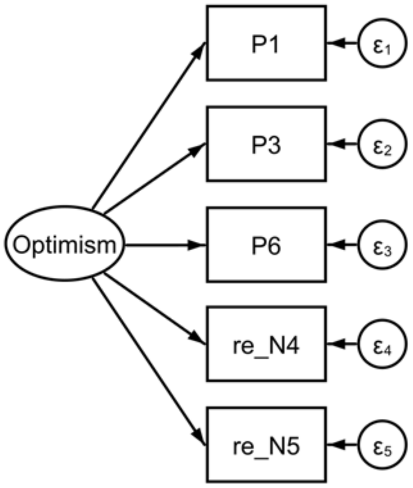

Model 1. Congeneric one-factor model

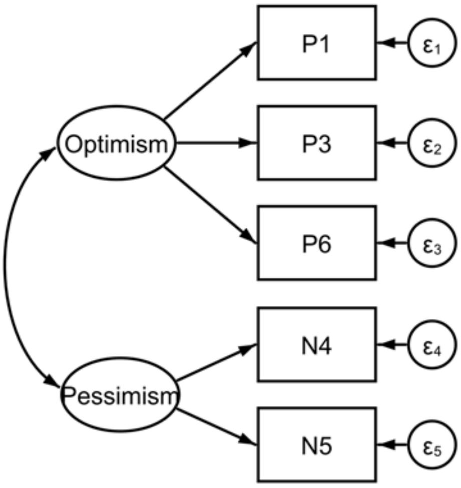

Model 2. Oblique two-factor model

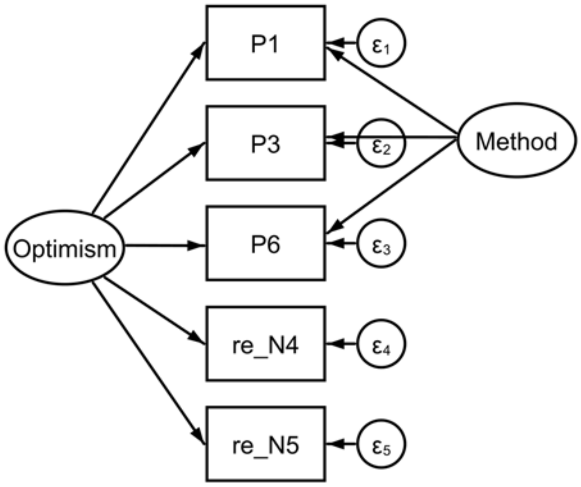

Model 3. One general factor (dispositional optimism) plus a method factor associated with positively worded items

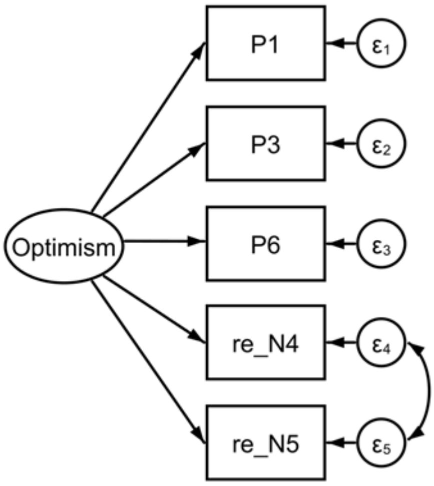

Model 4. One general factor (dispositional optimism) model with correlated errors between reversed negatively worded items

sFigure 3. Reassessed models for Life Orientation Test-Revised (five items).

Note. P1, P2, and P3 represent positively worded items 1, 3, and 6, respectively. N2, N4 and N5 represent negatively worded items 2, 4 and 5, respectively. re_N2, re_N4 and re_N5 represent reversed negatively worded items 2, 4 and 5, respectively.

Supplementary Content 2

Latent growth curve model

Overview

There are two ways to analyze longitudinal data, univariate and multivariate analysis. Latent growth curve (LGC) model is a multivariate analysis (in the sense that the data is in wide format) [1-4]. Mathematically, it is equivalent to mixed effects model, which is a univariate analysis (in the sense that the data is in long format) [1-4]. A potential advantage of the LGC model is that it can incorporate factor analysis to remove the influence of measurement error when including exposures [1-4]. In this study, dispositional optimism was measured by the Life Orientation Test-Revised (LOT-R), and based on the reliability analysis, roughly half of the variation in the observed LOT-R sum score could be attributed to measurement error. Therefore, we adopted LGC model in this study, and in the sensitivity analysis, we incorporated factor analysis into the LGC model to eliminate the influence of measurement error in LOT-R.

In the LGC model, there are two important parameters, intercept and slope [4]. The mean of intercept represents the average value of outcome at the starting time point, while the mean of slope represents the rate of change in the outcome over time [4]. Both the intercept and the slope are allowed to vary randomly across individuals to capture potential individual differences [4].

Analysis steps

In the present study, the first aim was to examine the trajectory of psychological distress from 1 year to 2 years after esophageal cancer surgery. The diagram for related LGC model is

displayed in Figure S1. The mean of the intercept represented the average log-odds of psychological distress at 1 year after surgery, while the mean of the slope referred to the average rate of change in the log-odds of psychological distress over time. This model accounts for individual variations in both the intercept and the slope parameters. Because the intercept is constant for all individuals across time, its loadings are fixed at "1". For the slope, we fixed its loadings on repeated measures as "0, 1, 2", such that we assumed that psychological distress increased linearly with time. Furthermore, we evaluated non-linear trajectory by adding another quadratic slope term and fixing its loadings on repeated measures as "0, 1, 4", but the results did not support non-linear trajectory. In addition, there was no statistically significant variation in the slope ($p = 0.305$). Therefore, we adopted a random intercept model in this study.

For the second aim (whether dispositional optimism predicted the risk of psychological distress), we regressed the intercept on dispositional optimism, with stepwise adjustment for known and potential confounders. In the main analysis, dispositional optimism was represented by LOT-R sum score. Related diagram is represented in Figure S2. In the sensitivity analysis, we incorporated factor analysis into the model and used latent factor to represent dispositional optimism, and Figure S3 displays related diagram.

Reference

1. Ghisletta P, Renaud O, Jacot N, Courvoisier D (2015) Linear Mixed-Effects and Latent Curve Models for Longitudinal Life Course Analyses. In: Burton-Jeangros C, Cullati S, Sacker A, Blane D (eds) A Life Course Perspective on Health Trajectories and Transitions. Cham (CH), pp 155-178. doi:10.1007/978-3-319-20484-0_8

2. Chou CP, Bentler PM, Pentz MA (1998) Comparisons of two statistical approaches to study growth curves: The multilevel model and the latent curve analysis. Structural Equation Modeling: A Multidisciplinary Journal 5 (3):247-266. doi:10.1080/10705519809540104

3. McNeish D, Matta T (2018) Differentiating between mixed-effects and latent-curve approaches to growth modeling. Behav Res Methods 50 (4):1398-1414. doi:10.3758/s13428-017-0976-5

4. Duncan TE, Duncan SC (2009) The ABC's of LGM: An Introductory Guide to Latent Variable Growth Curve Modeling. Soc Personal Psychol Compass 3 (6):979-991. doi:10.1111/j.1751-9004.2009.00224.x

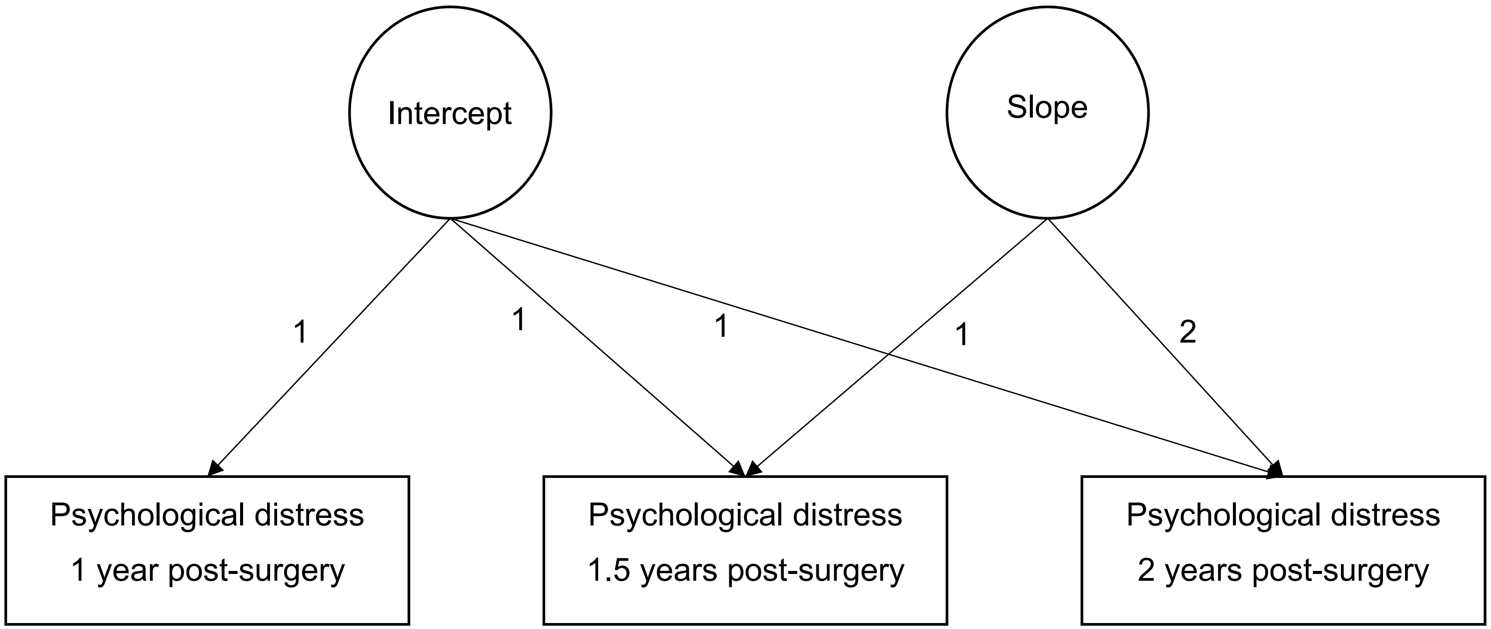

Figure S1. Diagram for the latent growth curve model examining the trajectory of psychological distress from 1 year to 2 years after esophageal cancer surgery

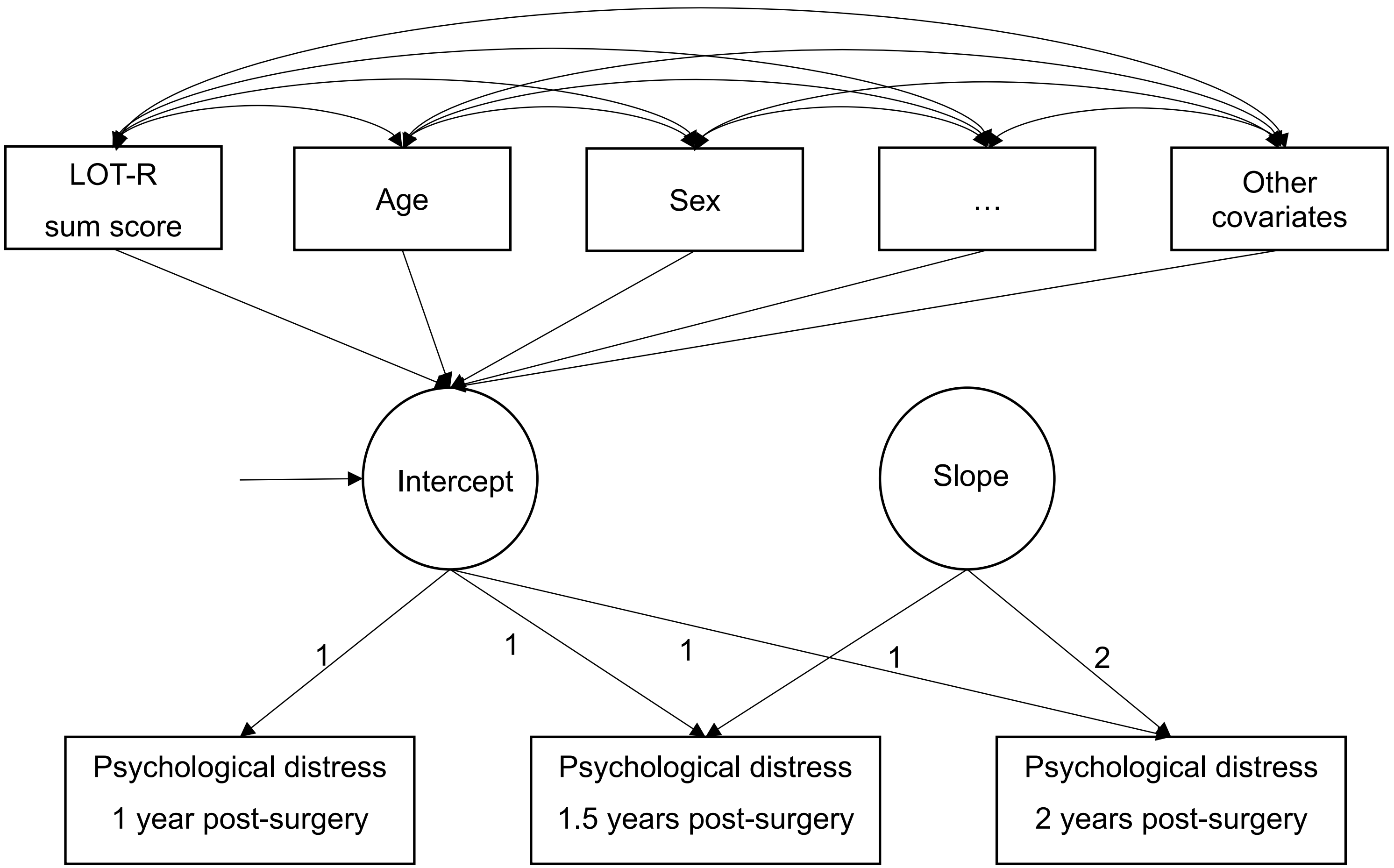

Figure S2. Diagram for the latent growth curve model in the main analysis (dispositional optimism is presented by LOT-R sum score) assessing the predictive effect of dispositional optimism on psychological distress after esophageal cancer surgery

Note. LOT-R: Life Orientation Test-Revised. Sum score of LOT-R: sum score of three positively worded items and two reversed negatively worded items.

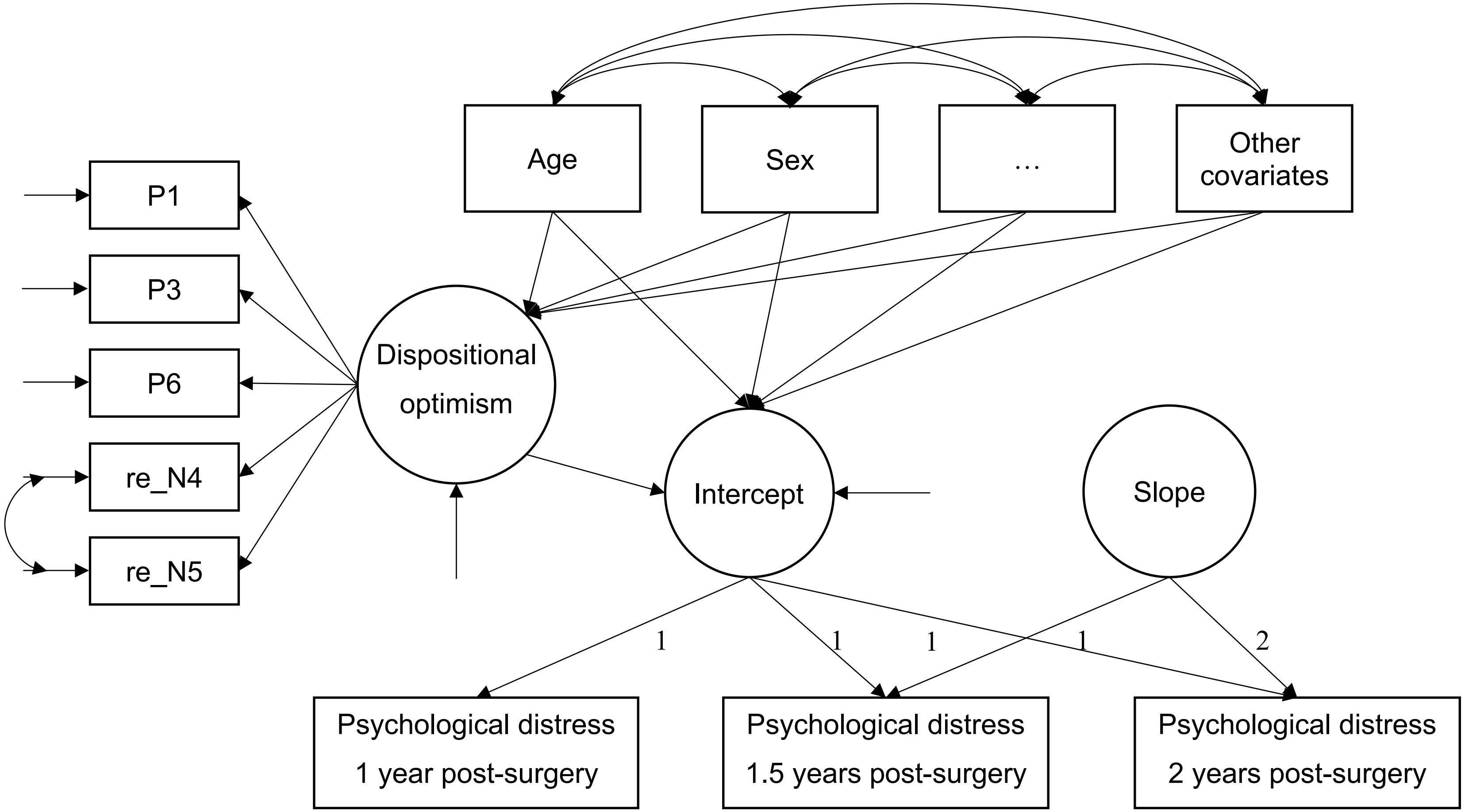

Figure S3. Diagram for the latent growth curve model in the sensitivity analysis (dispositional optimism is represented by a latent factor) assessing the predictive effect of dispositional optimism on psychological distress after esophageal cancer surgery

Note. P1, P2, P6, re_N4 and re_N5 are items in the Life Orientation Test-Revised. P1, P2, and P3 represent positively worded items 1, 3, and 6, respectively. re_N4 and re_N5 represent reversed negatively worded items 4 and 5, respectively.
